# Implementation and Evaluation of an Offline RPA-Based Scheduling Visualization Tool for Radiotherapy Under Security Constraints

**DOI:** 10.1007/s10916-025-02238-4

**Published:** 2025-08-07

**Authors:** Takuya Ito, Ryuhei Takeo

**Affiliations:** 1 Department of Radiotherapy Quality Management, Sanshikai Tomei Atsugi Hospital, Atsugi, Japan; 2 Department of Radiology, The University of Tokyo Hospital, Tokyo, Japan; 3 General Affairs Department, Sanshikai, Atsugi Japan

## Abstract

Timely access to radiotherapy appointment information remains challenging in hospitals—including many in Japan—due to fragmented information systems and strict security policies restricting access to databases or application programming interfaces (API). We developed an offline robotic process automation (RPA) workflow that captures scheduling data directly from the graphical user interfaces (GUIs) of the electronic medical record (EMR) and radiology information system (RIS) consolidating new-patient consultations, computed tomography (CT) simulations, and first-fraction irradiation sessions into a color-coded, four-week Excel calendar. The robot, scripted using the no-code KEYENCE RK-10 environment, logs into each application, applies rule-based filters, exports comma-separated-values (CSV) files, and populates a pre-formatted template without requiring any back-end modifications. During a two-week deployment at a community radiotherapy center, each weekday run finished in a median 3.9 min [inter-quartile range, IQR 3.1–4.8 min] and listed 36 appointments (16 consultations, five CTs, and 15 first-fraction irradiations). Two discrepancies compared to a legacy whiteboard revealed manual omissions. A semi-structured survey of 12 staff members recorded the highest Likert score [median 5.0, IQR 4.0–5.0] for reductions in documentation time, perceived workload, and error frequency in favor of the RPA implementation. This lightweight GUI-level approach provides a secure and rapidly deployable solution for small- to medium-sized radiotherapy centers operating under stringent information-technology (IT) policies, enhancing scheduling accuracy and staff efficiency without database integration. EMR, RPA, Radiation Therapy, Automation, Visualization.

**Supplementary Information:**

The online version contains supplementary material available at 10.1007/s10916-025-02238-4.

## Introduction

In Japan, radiotherapy scheduling often relies on manual methods like whiteboards and fragmented digital systems, contributing to omissions, duplicated efforts, and a lack of transparency [1–4]. In response to such fragmentation, national efforts have emphasized the development of standardized electronic medical record platforms and HL7 FHIR-based interoperability frameworks [2, 5].

At our institution, the electronic medical record (EMR) system (e-Karte, Software Service, Inc.) and the radiology information system (RIS; ACTRIS2, J-MAC SYSTEM) are connected, but the oncology information system (OIS; Mosaiq ver.2.83, Elekta) is used only as a record-and-verify system and is not integrated with either the EMR or RIS. Because only one Mosaiq client exists and is restricted to technical staff, clinical personnel cannot easily access up-to-date scheduling information.

In Japan, non-standardized EMR systems and strict information security policies limit access to direct databases and application programming interfaces (APIs), restricting the implementation of conventional information technology (IT) solutions in clinical settings [6, 7]. To address this, we developed a lightweight, secure robotic process automation (RPA) tool that extracts scheduling data from the graphical user interfaces (GUIs) and compiles it into a unified Excel calendar.

## Methods

### System Architecture

Our RPA tool was developed using the KEYENCE RK-10 no-code RPA software, which automates GUI-level interactions by replicating user mouse clicks and keystrokes, ensuring compatibility with hospital IT policies.

**Fig. 1:**
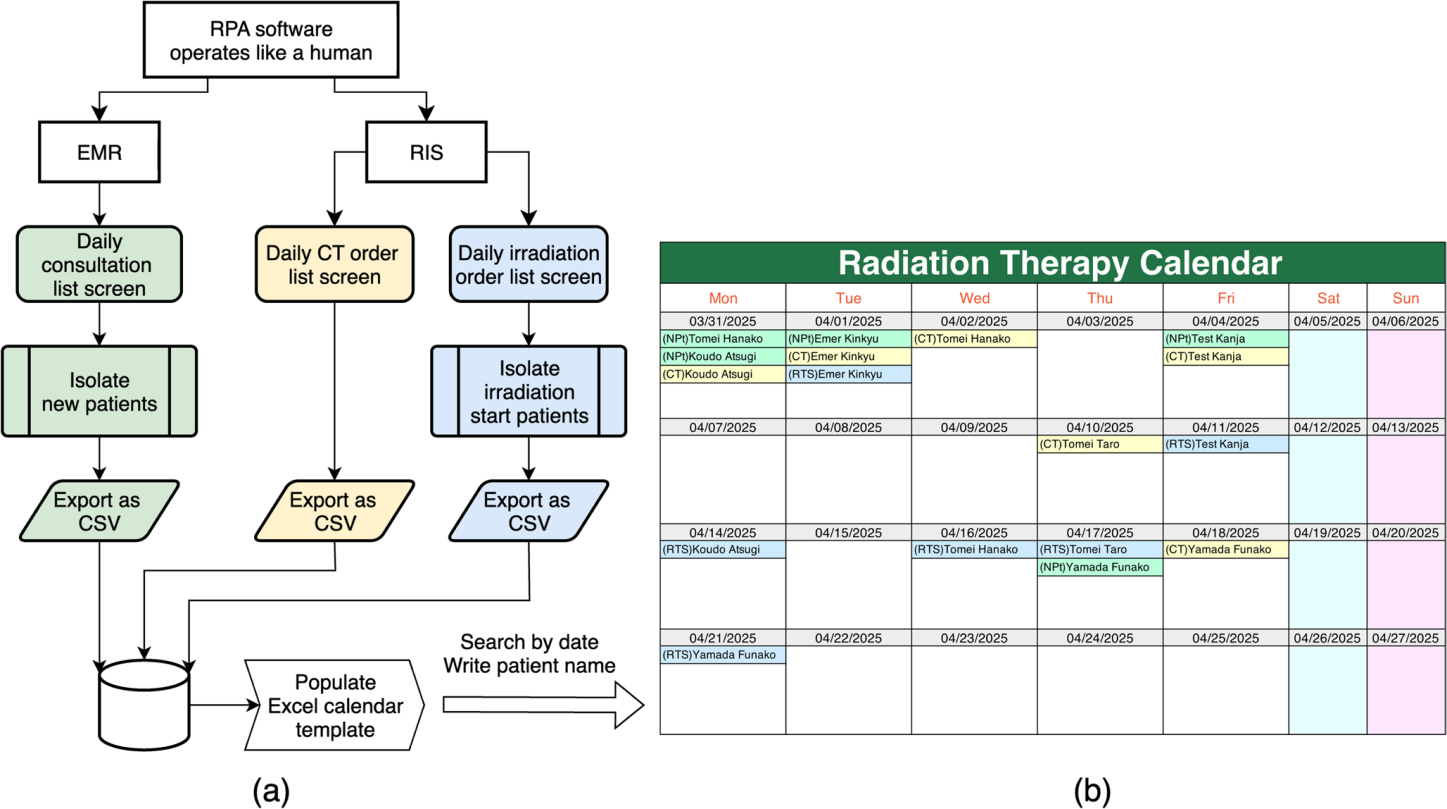
Workflow of the RPA-based tool for extracting scheduling information from EMR and RIS user interfaces and generating an Excel-based visual calendar

It executes the following sequence (Fig. 1(a)):

1. Log in to both the EMR and RIS systems in parallel.
2. Open the daily lists (consultations, planning-CT orders, irradiation orders).
3. Apply the isolation filters (see Table 1).
4. Export the filtered lists as CSV files.
5. Populate the structured Excel calendar template.
6. Display the updated calendar on the department’s large monitor.

Each weekday at 08:00 h, the robot initiates this workflow. After applying the isolation rules detailed in Table 1 (Step 3), the robot merges in memory the three CSV files generated in Step 4. To keep the calendar current, it first clears all entries in the Excel template and then writes the consolidated dataset (Step 5). Because our institution uses a dedicated radiotherapy CT scanner, coordination with the diagnostic radiology department was not required; the simple “RT-CT” modality filter shown in Table 1 was sufficient to isolate planning scans.

**Table 1:** Isolation rules used by the robot

Appointment Type	Source List	Primary Filter	Secondary Keywords^a^	Note
New-patient consultation	EMR reservation list	Booking template = Radiation Oncology New Patient	“first visit”, “new”	List includes returning patients; template/keywords isolate new patients
Planning CT simulation	RIS CT orders	Modality = RT-CT	-	Filters only radiotherapy-specific CT orders
Irradiation start (first fraction)	RIS irradiation orders	-	“initial”	List includes continuing fractions; keyword isolates first fraction

(a) Secondary keywords are evaluated only when the primary filter alone is insufficient.

The generated calendar displays four weeks of scheduled events, aligned with the current date. To enhance clarity, color coding is applied: green for new patient consultations, yellow for CT simulation appointments, and light blue for irradiation start dates. A sample calendar layout is shown in Fig. 1(b). While the figure uses English labels for illustration, the actual operational calendar used in the hospital is presented in Japanese.

The daily state-refresh process implicitly handles both cancellations and modifications. When a booking is deleted or rescheduled at the source—by front-desk staff for consultations or by physicians for CT and irradiation orders—it is absent from its original slot in the next data capture. Consequently, the appointment either vanishes from the refreshed calendar or moves to its new time slot, ensuring the display always mirrors the current EMR/RIS schedules. No historical diff is kept. Each run is logged as “success” or an error code, and staff can relaunch the robot manually if an error window appears.

### Clinical Context

The tool was deployed at a community-based radiotherapy center with a single linear accelerator (Elekta Synergy) and a small multidisciplinary team of physicians, radiation therapists, nurses, and medical physicists. Before RPA implementation, scheduling was managed via a handwritten whiteboard, with updates manually coordinated among staff.

## Evaluation Approach

We conducted a two-week operational evaluation during which all scheduled appointments were reviewed for accuracy and completeness using the RPA-generated calendar during daily morning briefings. Prior to implementation, omissions of patient names and appointment dates were commonly identified through manual cross-checking.

A semi-structured questionnaire was distributed to 15 clinical staff directly involved in the radiotherapy workflow (3 physicians, 5 nurses, and 7 radiotherapy technologists, including the department head). The questionnaire included 13 items across five sections: (1) estimated manual input time, (2) perceived error frequency, (3) workload burden, (4) changes in efficiency and errors post-implementation, and (5) free-text suggestions for improvement. The survey, offered in both paper and online formats, combined Likert-type scales with one open-ended question. Participation was voluntary and anonymous. Respondents were selected by departmental heads based on their involvement in scheduling tasks; the authors were not involved in the selection process. Ethical approval was obtained from the institutional review board (Approval No. 2025002).

For analysis, time-range responses from the questionnaire (e.g., “5–10 min”) were converted to their mid-points (e.g., 7.5 min). All quantitative results are reported as medians with inter-quartile ranges (IQR).

Each item was rated on a 5-point ordinal scale as follows:

Whiteboard writing time (for each appointment time):

1 = < 5 min, 2 = 5–10 min, 3 = 10–15 min, 4 = 15–30 min, 5 = > 30 min.

Error frequency:

1 = Frequently (> every 2 weeks), 2 = Monthly, 3 = Every 2–3 months, 4 = < every 6 months, 5 = Never (0/year).

Perceived burden:

1 = Very burdensome, 2 = Somewhat burdensome, 3 = Neutral, 4 = Not very burdensome, 5 = Not burdensome at all.

Perceived change after RPA introduction (efficiency, error rate, and workload):

1 = Significantly increased, 2 = Somewhat increased, 3 = No change, 4 = Somewhat reduced/decreased, 5 = Significantly reduced/decreased.

## Results

### System Output

The RPA tool operated successfully without requiring backend modifications or additional software. It launches automatically at 08:00 h each weekday, with each run completed in a median of 3.9 min [IQR: 3.1–4.8 min] (*n* = 10). During the two-week evaluation, it accurately captured 36 radiotherapy-related appointments—16 new-patient consultations, 5 treatment-planning CTs, and 15 first-fraction irradiation sessions—and posted them to the calendar. In contrast, two omissions (one new-patient consultation and one first-fraction irradiation) were discovered on the handwritten whiteboard during morning briefings.

### Survey Outcomes (12 responses collected)

Twelve valid responses were received (physicians 3/3, nurses 5/5, radiotherapy technologists 4/7), yielding an overall response rate of 80%. Two additional responses were received after the deadline and were excluded from analysis.

Whiteboard writing time:

The median time required to manually enter information was 7.5 min [IQR: 2.5–7.5 min] for new patient appointments, 7.5 min [2.5–7.5 min] for CT simulations, and 7.5 min [7.5–7.5 min] for first-fraction irradiations.

Error frequency (before RPA):

Median error frequency scores were 3.5 [IQR: 3.0–4.0] across all three appointment types.

Perceived burden (before RPA):

Median burden scores were 3.0 [IQR: 2.0–3.2] across all three appointment types.

Perceived changes after RPA introduction:

Median Likert scores indicated perceived reductions in time, error frequency, and workload burden, all rated 5.0 [IQR: 4.0–5.0].

Suggestions:

Respondents suggested adding automated screen magnification, displaying treatment site information alongside patient names, and increasing data refresh frequencies.

## Discussion

We implemented a GUI-level RPA solution that runs on existing EMR interfaces to automatically generate an Excel calendar, requiring no infrastructure changes and complying with hospital security policies.

Prior to RPA implementation, staff estimated that manually transcribing three key appointment types required a cumulative total of 22.5 min. In contrast, the automated RPA tool completed the entire workflow in a median time of 3.9 min before work hours, eliminating the need for manual transcription during clinical operations. Although the present evaluation lacked pre-implementation quantitative data, the large differential between estimated manual effort and measured robot runtime supports a substantive efficiency gain. Manual transcription of appointments by both dedicated and non-dedicated staff was replaced with a scheduled RPA process, consolidating potential errors into a single automated checkpoint, which may suggest reduced omissions and cognitive load among staff.

Comprehensive systems such as ARIA Care Paths and Mosaiq QCLs enhance workflow management and quality assurance [8], but are costly and complex to implement. Custom solutions using EMR or OIS database APIs can enable advanced dashboards [9], but require standardized architectures and dedicated IT support. In contrast, our RPA-based approach operates without any system modifications and remains practical and accessible for small to medium-sized facilities under strict security constraints.

Although our formal evaluation covered only the initial two-week period, the robot has remained in daily use for more than three months, with all staff adopting the tool and retiring the manual whiteboard. During this period, no scheduling omissions have been reported at the morning conferences, and the calendar continues to be generated within the same few-minute runtime, indicating that the initial gains in accuracy and efficiency have been sustained. In RE-AIM terms, these real-world observations demonstrate lasting adoption and maintenance.

## Limitations

A key limitation is that our evaluation, being retrospective and confined to a two-week pilot, relied on subjective questionnaire data rather than objective metrics. Additionally, the small sample size (*n* = 12) limits statistical power, and the comparison between estimated manual task times and measured RPA runtime represents a methodological constraint requiring prospective validation. Staff selection by departmental heads based on scheduling involvement may introduce selection bias, though this reflects realistic adoption scenarios. Future prospective studies must capture quantitative indicators over extended periods to validate findings and assess generalizability across institutions.

The tool also depends on stable EMR/RIS screen layouts and CSV-export functions; major UI revisions would require reprogramming. Furthermore, the rule-based filters detailed in Table 1 are tailored to our institution’s specific software and operational conventions, and thus are not directly portable to other facilities without modification. More importantly, it captures only a subset of the radiotherapy workflow—consultation, planning-CT, and first-fraction scheduling—while other key workflow steps (e.g., contour delineation, plan creation and approval, patient-specific QA), which are currently managed by a separate paper-based checklist, remain outside its current scope. Extending coverage to the entire workflow or integrating with interactive scheduling platforms while preserving security compliance, is therefore a priority for subsequent development.

## Future Directions

While the current tool focuses on visualizing key appointments, its underlying approach has three strategic avenues for future development.

First, beyond simple visualization, the structured data captured daily by the RPA can be archived to create a longitudinal dataset of departmental workflow. Analyzing this data would enable the identification of operational bottlenecks and support predictive resource allocation.

Second, the tool’s scope can be expanded to include other key workflow events, such as treatment planning deadlines and quality assurance checks, to provide a more holistic view of the patient journey. While the current Excel-based calendar has limitations, the core RPA approach is applicable to broader contexts. Indeed, its potential for inter-departmental use has been noted, with possible applications in optimizing nurse staffing for contrast-enhanced imaging or developing a similar calendar for the endoscopy center.

Finally, while multi-center deployment by the authors is not feasible, generalizing the approach is a key objective. Enhancing the flexibility of the rule-based engine, as exemplified by Table 1, is crucial for adapting the tool to other clinical departments and potentially other institutions with different workflows.

## Conclusion

Our GUI-level RPA calendar securely visualized radiotherapy appointments under strict IT constraints and was perceived by staff to improve workflow efficiency and reduce time burden. Future work should quantify these gains and assess adaptability across diverse clinical settings.

## Supplementary Information

Below is the link to the electronic supplementary material.

Supplementary Material 1

Supplementary Material 2

## Data Availability

The de-identified staff-survey dataset that supports the findings of this study is openly available as a read-only Google Spreadsheet at https://docs.google.com/spreadsheets/d/1d_JuTnWu57WsRIuFQ1_aiiok-WqcZBcqgTAUradBmmY/edit? usp=sharing(accessed 19 June 2025).Variable definitions—including Likert-scale coding and item wording—are provided in the Methods section of the manuscript. An English version of the questionnaire is supplied as Supplementary File (Questionnaire_Eng.docx). Further clarifications are available from the corresponding author upon reasonable request.
