# Supplementary material for: Implementation and Evaluation of an Offline RPA-Based Scheduling Visualization Tool for Radiotherapy Under Security Constraints
Source: J Med Syst. 2025 Aug 7;49(1):102. doi: 10.1007/s10916-025-02238-4 (PMC12328462; doi:10.1007/s10916-025-02238-4)
Supplement: Supplementary file 1 — Supplementary Material 1 [file 10916_2025_2238_MOESM1_ESM.docx]

## Questionnaire on the effectiveness of the RPA calendar

## Department of Radiotherapy Quality Management Takuya Ito

## [Survey Assumptions]

RPA Calendar is a system that automatically collects patient names and schedules from electronic medical records and displays them as a calendar on the monitor.
The purpose of this survey is to evaluate the effectiveness of the RPA calendar and to verify its operational efficiency.

- Responses are voluntary, and there will be no disadvantages for not responding.
- Responses will be anonymous and no personally identifiable information will be collected.
- Responses submitted cannot be withdrawn or modified.

All 13 questions are optional. You may answer only some of them.

**Purpose of the Survey Results**

Survey results will be used only for the following purposes. They will not be used for any other purpose.
If you agree with the purpose of use, please check the appropriate box.

1. To report work-replacement time to the Administration Department.

**□ Agree　　　　　□ Disagree**

2. To be used for research and presentations.

**□ Agree　　　　　□ Disagree**


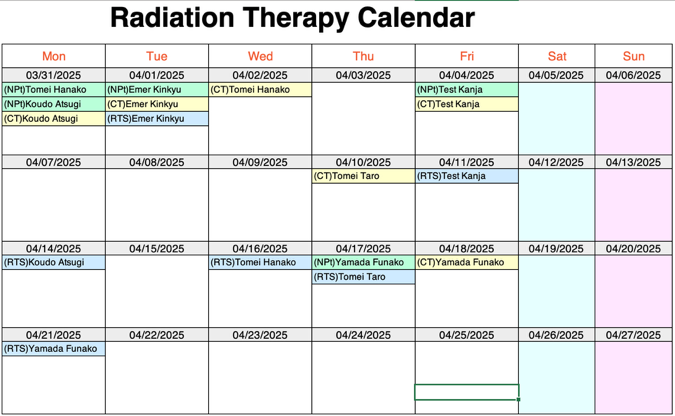

Image of RPA calendar

(The date of the first appointment, the date of CT scan, and the date of irradiation start will be included.)


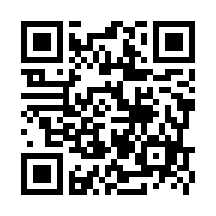


If you prefer to answer from Google Forms,

please scan this QR code.

### [Respondent Information]（Answers are optional）

- Occupation：☐ Physician ☐ Nurse ☐ Radiological Technologist
   ☐ Others（_________________）
- Experience with RPA calendar system:
  ☐ Less than 1 week ☐ 1-2 weeks ☐ more than 2 weeks
- Years of experience in patient schedule verification and filling out：
  _____ Years

## [1. Questions about work time: Before RPA introduction] Please answer as if it were your own work. Please answer based on your own experience. (If you have no or little experience, please answer by estimation.) (Our average performance is 15 each for “new patients,” “planned CT,” and “irradiation start” in 3 weeks.)

### It is intended to be used for morning conferences.

### New patients should be listed on the whiteboard as “today's” patients at the morning conference. This will be repeated over a 3-week period, for a total of 15 patients to be listed.

### The planned CT and irradiation start will be listed on the whiteboard for the next 3 weeks (15 patients total) and added to the whiteboard as new appointments are added.

### Q1. How much time does it take to write the names and dates of new patients on the whiteboard? (e.g., writing the information for the next day in advance)

- Less than 5 minutes
- 5–10 minutes
- 10–15 minutes
- 15–30 minutes
- More than 30 minutes

### Q2. How much time does it take to write the names and dates of CT simulation patients on the whiteboard?

- Less than 5 minutes
- 5–10 minutes
- 10–15 minutes
- 15–30 minutes
- More than 30 minutes

### Q3. How much time does it take to write the names and dates of irradiation start patients on the whiteboard?

- Less than 5 minutes
- 5–10 minutes
- 10–15 minutes
- 15–30 minutes
- More than 30 minutes

## [2. Questions about error rates: Before RPA introduction]

## **We have about 250 radiotherapy patients per year, and we also have about 250 new patients, planned CT, and irradiation starts each.** Please answer as if it were your own work. Please answer based on your own experience. (If you have no or little experience, please answer by estimation.)

In the following questions, “error” refers to cases where the information written on the whiteboard, which should be confirmed by all members during the morning conference, is incorrect.
Specifically, this includes the following:

### • Omission: A patient who should have been listed is not written. • Incorrect Recording: The patient’s name or date is written incorrectly.

### Q4. How often do you think errors occur when writing the names and dates of new patients on the whiteboard for the next day?

- Never (0 incidents per year)
- Very rarely (less than once every six months)
- Occasionally (about once every 2–3 months)
- Relatively frequently (about once a month)
- Frequently (more than once every two weeks)

### Q5. How often do you think errors occur when writing the names and dates of CT simulation patients on the whiteboard? (For patients with confirmed CT simulation schedules)

- Never (0 incidents per year)
- Very rarely (less than once every six months)
- Occasionally (about once every 2–3 months)
- Relatively frequently (about once a month)
- Frequently (more than once every two weeks)

### Q6. How often do you think errors occur when writing the names and dates of irradiation start patients on the whiteboard or other surfaces? (For patients with confirmed irradiation schedules)

- Never (0 incidents per year)
- Very rarely (less than once every six months)
- Occasionally (about once every 2–3 months)
- Relatively frequently (about once a month)
- Frequently (more than once every two weeks)

## [3. Questions about Workload: Before RPA introduction] Please answer as if it were your own work. Please answer based on your own experience. (If you have no or little experience, please answer by estimation.)

### Q7. How burdensome do you feel the task of writing the names and dates of new patients on the whiteboard is?

- Not burdensome at all
- Not very burdensome
- Neutral
- Somewhat burdensome
- Very burdensome

### Q8. How burdensome do you feel the task of writing the names and dates of CT simulation patients on the whiteboard is?

- Not burdensome at all
- Not very burdensome
- Neutral
- Somewhat burdensome
- Very burdensome

### Q9. How burdensome do you feel the task of writing the names and dates of irradiation start patients on the whiteboard or other surfaces is?

- Not burdensome at all
- Not very burdensome
- Neutral
- Somewhat burdensome
- Very burdensome

## [4. Effectiveness of RPA Calendar] Please answer based on your own experience. (If you have no or little experience, please answer by estimation.)

### Q10. How has the time required for schedule checking and writing changed with the introduction of the RPA calendar?

- Significantly reduced
- Somewhat reduced
- No change
- Somewhat increased
- Significantly increased

### Q11. How has the occurrence of omissions or miswriting changed with the introduction of the RPA calendar?

- Significantly decreased
- Somewhat decreased
- No change
- Somewhat increased
- Significantly increased

### Q12. How has the burden of schedule management changed with the introduction of the RPA calendar?

- Significantly reduced
- Somewhat reduced
- No change
- Somewhat increased
- Significantly increased

[5. Comments]

### Q13. Please provide any additional comments or suggestions for improvement regarding the RPA calendar.
