# Supplementary material for: Implementation and Evaluation of an Offline RPA-Based Scheduling Visualization Tool for Radiotherapy Under Security Constraints
Source: J Med Syst. 2025 Aug 7;49(1):102. doi: 10.1007/s10916-025-02238-4 (PMC12328462; doi:10.1007/s10916-025-02238-4)
Supplement: Supplementary file 2 — Supplementary Material 2 [file 10916_2025_2238_MOESM2_ESM.docx]

## RPAカレンダーの効果に関するアンケート

## 放射線治療品質管理室　伊藤拓也

## 【アンケートの前提】

RPAカレンダーは電子カルテから患者名やスケジュールを自動的に収集し、モニターにカレンダーとして表示するシステムです。
このアンケートは、RPAカレンダーの効果を評価し、業務効率化を検証することを目的としています。
・回答は任意であり、回答しないことによる不利益はありません。
・回答は匿名で集計され、個人を特定できる情報は収集しません。
・提出した回答は撤回や修正ができません。

全13問の質問は任意で回答できます。回答は一部のみでも構いません。

**アンケート結果の利用目的**
アンケート結果は以下の目的にのみ使用されます。それ以外の目的には使用されません。
利用目的に同意する場合は、その項目にチェックを入れてください。

1.業務代替時間を管理部に報告するため。

**□ 同意します　　　　　□ 同意しません**

2.研究および発表に使用するため。

**□ 同意します　　　　　□ 同意しません**


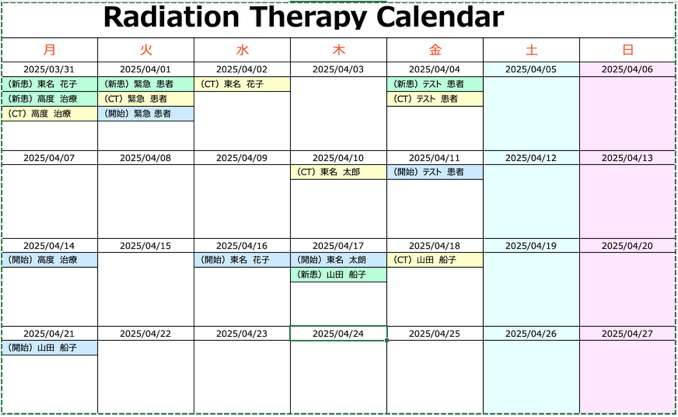
　　　　　
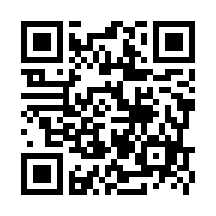


RPAカレンダーイメージ図

（初診日、CT撮影日、照射開始日が載ります）　　　 Google Formsから
回答する場合はこちら

### 【回答者情報】（回答は任意です）

- 職種：☐ 医師 ☐ 看護師 ☐ 診療放射線技師 ☐ その他（_________________）
- RPAカレンダーシステムの利用経験：☐ 1週間未満 ☐ 1〜2週間 ☐ 2週間以上
- 患者スケジュール確認および記入作業の経験年数：_____ 年

## 【1. 作業時間に関する質問：RPA導入前】 自分の業務であるとしてお答えください。 ご自身の経験でお答えください。経験が無い方や少ない方は推定で良いのでお答えください。 当院の平均的な実績は3週間で「新患」「計画CT」「照射開始」がそれぞれ15人です

### 朝のカンファレンスに使うことを前提としています。 ・新患は、朝のカンファレンスで「当日分」の患者がホワイトボードに記載されていれば良いです。これを3週間にわたり繰り返し、合計15人を記載することになります。 ・計画CTおよび照射開始は、今後3週間分の予定（合計15人分）をホワイトボードに記載し、新たな予定が追加されるたびに追記します。

### Q1.新患の氏名と日付をホワイトボードに記載するのにかかる合計時間はどのくらいですか？（前日などに翌日分を書く）

- 5分未満
- 5〜10分
- 10〜15分
- 15〜30分
- 30分以上

### Q2.計画CT患者の氏名と日付をホワイトボードに記載するのにかかる合計時間はどのくらいですか？

- 5分未満
- 5〜10分
- 10〜15分
- 15〜30分
- 30分以上

### Q3.照射開始患者の氏名と日付をホワイトボード等に記載するのにかかる合計時間はどのくらいですか？

- 5分未満
- 5〜10分
- 10〜15分
- 15〜30分
- 30分以上

## 【2. エラー率に関する質問：RPA導入前】

## **当院は年間250人程度の放射線治療患者がおり、新患、計画CT、照射開始もそれぞれ250件程度です。** 自分の業務であるとしてお答えください。 ご自身の経験でお答えください。経験が無い方や少ない方は推定で良いのでお答えください

### 以下の質問で「エラー」とは、朝のカンファレンス時に全員が確認するホワイトボードに記載されるべき情報が事実と異なる場合を指します。 具体的には以下が含まれます：

### •記載漏れ：本来書かれるべき患者が記載されていない •誤記載：患者氏名または日付が誤って記載されている

### Q4.新患の記載でエラーはどのくらいの頻度で発生すると考えますか？（翌日の新患をホワイトボードに書く）

- 全く発生しない（0件/年間）
- ごくまれに発生する（半年に1回以下）
- 時々発生する（2〜3か月に1回程度）
- 比較的頻繁に発生する（1か月に1回程度）
- 頻繁に発生する（2週間に1回以上）

### Q5. 計画CT患者の記載において、エラーはどのくらいの頻度で発生すると考えますか？（予定が決定している計画CT患者を書く）

- 全く発生しない（0件/年間）
- ごくまれに発生する（半年に1回以下）
- 時々発生する（2〜3か月に1回程度）
- 比較的頻繁に発生する（1か月に1回程度）
- 頻繁に発生する（2週間に1回以上）

### Q6. 照射開始患者の記載において、エラーはどのくらいの頻度で発生すると考えますか？（照射が決定した患者を書く）

- 全く発生しない（0件/年間）
- ごくまれに発生する（半年に1回以下）
- 時々発生する（2〜3か月に1回程度）
- 比較的頻繁に発生する（1か月に1回程度）
- 頻繁に発生する（2週間に1回以上）

## 【3. 作業負担度：RPA導入前】 自分の業務であるとしてお答えください。 ご自身の経験でお答えください。経験が無い方や少ない方は推定で良いのでお答えください

### Q7. 新患の氏名と日付をホワイトボードに記載する作業は、どの程度負担に感じますか？

- 全く負担に感じない
- あまり負担に感じない
- どちらでもない
- やや負担に感じる
- 非常に負担に感じる

### Q8. 計画CT患者の氏名と日付をホワイトボードに記載する作業は、どの程度負担に感じますか？

- 全く負担に感じない
- あまり負担に感じない
- どちらでもない
- やや負担に感じる
- 非常に負担に感じる

### Q9. 照射開始患者の氏名と日付をホワイトボードに記載する作業は、どの程度負担に感じますか？

- 全く負担に感じない
- あまり負担に感じない
- どちらでもない
- やや負担に感じる
- 非常に負担に感じる

## 【4. RPAカレンダーの効果】 ご自身の経験でお答えください。経験が無い方や少ない方は推定で良いのでお答えください

### Q10. RPAカレンダーの導入により、スケジュール確認と記載の作業時間はどのように変化しましたか？

- 大幅に短縮された
- ある程度短縮された
- 変わらない
- ある程度増加した
- 大幅に増加した

### Q11. RPAカレンダーの導入により、記載漏れや誤記載はどのように変化しましたか？

- 大幅に減少した
- ある程度減少した
- 変わらない
- ある程度増加した
- 大幅に増加した

### Q12. RPAカレンダーの導入により、スケジュール管理の負担はどのように変化しましたか？

- 大幅に減少した
- ある程度減少した
- 変わらない
- むしろ負担が増えた
- 大幅に増加した

【5. コメント欄】

### Q13. その他RPAカレンダーに関するご意見や改善点があればご記入ください
